# Relationship Obsessive–Compulsive Disorder: Interference, Symptoms, and Maladaptive Beliefs

**DOI:** 10.3389/fpsyt.2016.00058

**Published:** 2016-04-18

**Authors:** Guy Doron, Danny Derby, Ohad Szepsenwol, Elad Nahaloni, Richard Moulding

**Affiliations:** ^1^Department of Psychology, Interdisciplinary Center (IDC) Herzliya, Herzliya, Israel; ^2^Cognetica – The Israeli Center for Cognitive Behavioral Therapy, Tel Aviv, Israel; ^3^Department of Psychology, University of Minnesota, Minneapolis, MN, USA; ^4^Centre for Mental Health and Wellbeing Research (CMHWR), School of Psychology, Deakin University, Burwood, VIC, Australia

**Keywords:** relationship obsessive–compulsive disorder (ROCD), OCD and anxiety disorders, cognitive therapy, maladaptive beliefs, relationships

## Abstract

**Background:**

Obsessive preoccupation, doubts, and compulsive behaviors focusing on one’s romantic relationship and partner are receiving increasing clinical, theoretical, and empirical attention. Commonly referred to as relationship obsessive–compulsive disorder (ROCD), such symptoms have been linked with decreased relational and sexual functioning and lower mood, even after controlling for other obsessive–compulsive disorder (OCD) symptoms. To date, however, these symptoms have been studied in community samples alone. In the present study, we compared levels of interference, OCD, and mood symptoms between clinical participants with ROCD, OCD, and community controls. We also examined group differences in maladaptive beliefs previously linked with OCD and ROCD.

**Method:**

Participants included 22 ROCD clients, 22 OCD clients, and 28 community controls. The Mini International Neuropsychiatric Interview was used to attain clinical diagnoses of OCD and ROCD. The Yale–Brown Obsessive–Compulsive Scale was used to evaluate primary-symptoms severity. All participants completed measures of symptoms and dysfunctional beliefs.

**Results:**

ROCD clients reported more severe ROCD symptoms than the OCD and control groups. ROCD and OCD clients did not differ in severity of their ­primary-symptoms. ROCD clients scored higher than the other groups on maladaptive OCD-related and relationship-related beliefs. Finally, ROCD clients showed more severe depression symptoms than community controls.

**Conclusion:**

ROCD is a disabling presentation of OCD that warrants research attention. Maladaptive OCD-related and relationship-related beliefs may be implicated in the development and maintenance of ROCD.

## Introduction

Obsessive–compulsive disorder (OCD) is a disabling disorder comprising various symptom dimensions including contamination fears, repugnant aggressive, sexual or blasphemous thoughts, and compulsive behaviors such as washing, checking, and ordering [e.g., Ref. (1)]. One understudied OCD symptom dimension receiving increasing research and clinical attention involves obsessive–compulsive (OC) symptoms focused on close interpersonal relationships [e.g., Ref. (2–6)]. Commonly referred to as relationship obsessive–compulsive disorder (ROCD), this OCD presentation has been associated with significant personal and relational consequences [see Ref. (2) for a review]. To date, however, no study has systematically compared clinical samples of individuals with ROCD, OCD, and non-clinical controls on levels of functioning, OC symptoms, mood, and maladaptive beliefs.

### Relationship Obsessive–Compulsive Disorder

Relationship obsessive–compulsive disorder often involves doubts and preoccupation centered on the perceived suitability of the relationship itself including the strength of one’s feelings toward their partner, the “rightness” of the relationship and the partner’s feelings toward oneself. Such symptoms have been referred to as *relationship-centered* OC symptoms (5). Relationship-centered obsessions have been theoretically and empirically differentiated from worries (2, 4). For instance, relationship-centered symptoms are less self-congruent, more likely to be associated with compulsive behaviors, and are perceived as less rational than worries. Furthermore, whereas worries commonly appear in verbal format and pertain to a variety of life domains, relationship-centered obsessions come in a variety of forms, including images, thoughts, and urges and focus on the relationship domain. Indeed, recently relationship-centered symptoms were found to correlate only moderately with worries as assessed by the Penn State Worry Questionnaire [*r* = 0.21 (4)].

Another common ROCD presentation involves disabling preoccupation with perceived deficits of the relationship partner in a variety of domains such as appearance, intelligence, sociability, and morality. This ROCD presentation has been coined *partner-focused* OC symptoms (5, 6). Although similar in some ways to what has been referred to in the literature as Body Dysmorphic Disorder by Proxy [i.e., obsessional focus on perceived physical flaws; see Ref. (7)], partner-focused OC symptoms refer to obsessional preoccupation with a wider variety of the partner’s flaws (2).

Relationship obsessive–compulsive disorder symptoms often come in the form of thoughts (e.g., “Is he the right one?”) and images (e.g., face of the relationship partner), but can also occur in the form of urges (e.g., to leave one’s current partner). Such intrusions are generally ego-dystonic, as they contradict the individual’s personal values (e.g., “appearance should not be important in selecting a relationship partner”) and/or subjective experience of the relationship (e.g., “I love her, but I can’t stop questioning my feelings”). Hence, they are perceived as unacceptable and unwanted by the individual, and often bring about feelings of guilt and shame regarding the occurrence and/or content of the intrusions. Compulsive behaviors in ROCD may include repeated monitoring of one’s own feelings, comparisons of partner’s characteristics with those of other potential partners, neutralizing (e.g., visualizing being happy together), and reassurance seeking. These compulsive behaviors are aimed at alleviating the significant distress caused by the unwanted intrusions (2).

Recent studies in community cohorts have shown ROCD symptoms are associated with severe personal and dyadic distress. ROCD symptoms were linked with other OCD symptoms, negative affect, low self-esteem, low relationship satisfaction, attachment insecurities, and impaired sexual functioning (3, 5, 8). Moreover, ROCD symptoms significantly predicted relationship dissatisfaction and depression over-and-above common OCD symptoms and other mental health and relationship insecurity measures (4, 5, 8).

### General and Specific Beliefs Associated With ROCD

Cognitive-behavioral theories of OCD suggest that the catastrophic (mis)interpretation of normal internal or external stimuli (e.g., intrusive thoughts) are causal to the onset and maintenance of OCD (9). Catastrophic appraisals of such stimuli promote selective attention and ineffective strategies when responding to their occurrence, which paradoxically exacerbate their frequency and emotional impact [e.g., compulsive behaviors (10–12)].

Findings from community participants show OCD-related beliefs are associated with ROCD symptoms (5, 8). Attributing exaggerated importance to the mere occurrence of thoughts, for instance, may increase attention to common relationship doubts and promote the use of ineffective, counterproductive thought suppression strategies. However, the moderate magnitude of the correlations found between ROCD symptoms and OCD-related maladaptive beliefs suggests that other cognitive biases also contribute to the development and maintenance of relationship-related OC phenomena (5, 8).

Recently, Doron and colleagues (2, 5, 8) proposed that catastrophic beliefs regarding future consequences of relationship-related decisions may be germane to the development and maintenance of ROCD. Following Rachman’s OCD model (12), they proposed several beliefs that are likely to promote distress following the occurrence of common relationship concerns. These include catastrophic beliefs regarding the consequence of remaining in a “wrong” relationship (e.g., wrong romantic decision would put me on a path of great misery) or of leaving an existing relationship (e.g., breaking up with my partner might cause irreparable damage). Thus, maladaptive OCD-related and specific relationship beliefs were proposed to be implicated in the exacerbation of common relationship concerns into debilitating obsessions.

### The Current Study

The current investigation is the first study comparing patterns of OC and affective symptoms, OCD-related beliefs, and relationship-related maladaptive beliefs between clinical OCD, ROCD, and community samples. Non-clinical participants experience OCD-related phenomena and associated cognitions [e.g., Ref. (13)]. They may differ from clinical participants, however, in severity and symptom-related impairment. Furthermore, extending the analysis to clinical participants will enable stronger inferences regarding the importance and specificity of OCD-related beliefs and maladaptive relational beliefs to ROCD.

We made several hypotheses: first, we hypothesized that clients identified as presenting with OCD with a romantic relationship theme (either relationship-centered or partner-focused) using the MINI interview would show higher relationship-centered and partner-focused OC symptom using self-report measures than would OCD clients and community controls. Second, we expected ROCD and OCD clients would show similar levels of functioning, distress, resistance attempts, and degree of control related to their primary obsessions and compulsions. Third, we hypothesized that ROCD and OCD clients would show higher levels of OC and depressive symptoms than community controls. Finally, we hypothesized that ROCD clients would report more maladaptive relational beliefs than both OCD clients and community controls, with both clinical groups reporting more OCD-related beliefs than community controls.

## Materials and Methods

### Participants

Participants in the OCD and ROCD groups were recruited via psychology clinics in Israel, and advertisements in the local paper to take part in an OCD study. Diagnoses were confirmed using the Mini international neuropsychiatric interview [MINI PLUS version 5.0 (14)] administered by registered psychologists, who had received prior training in MINI administration. Entry criteria for inclusion in the study were: (a) a primary diagnosis of OCD (OCD group) or ROCD, (b) no current substance abuse, and (c) no current or past schizophrenia, bipolar disorder, or organic mental disorder.

The OCD group included 22 participants with a primary diagnosis of OCD (13 females; age ranging from 18 to 49, *M* = 29.43, SD = 8.33). Four of the participants presented with a concurrent diagnosis of major depressive disorder (MDD). Eight participants presented with a secondary diagnosis of one or more anxiety disorders [*n* = 4 social phobia; *n* = 5 Generalized Anxiety Disorder (GAD); *n* = 2 Panic Disorder; *n* = 3 Specific Phobia]. Sixty-four percent (*n* = 14) of individuals in the OCD group received anti-depressant or anxiolytic medication at the time of the study. Forty-six percent (*n* = 10) were in a romantic relationship.

The ROCD group included 22 participants with a primary diagnosis of OCD with a relationship theme (nine females; age 21–40, *M* = 29.89, SD = 4.76). Four participants presented with concurrent MDD and four with a secondary diagnosis of OCD (other than ROCD). Five participants in this group presented with a secondary diagnosis of one or more anxiety disorders (*n* = 3 Specific Phobia, *n* = 1 Social Anxiety Disorder; *n* = 3 GAD). Fifty-five percent (*n* = 12) of individuals in the ROCD group received anti-depressant or anxiolytic medication at the time of the study. Eighty-two percent (*n* = 18) were in a romantic relationship.

The community control group included 28 participants (17 females; age ranging from 18 to 57, *M* = 31.50, SD = 8.90). Eighty-nine percent of them (*n* = 25) were in a romantic relationship at the time of the study. These participants were a random sample of a larger Israeli community group participating in another study. Exclusion criteria entailed a current psychiatric disorder, drug abuse, or current psychiatric treatment. No ­significant age (*F* < 1) or gender [χ^2^ (df) = 2.26, ns] differences were found between the three groups.

### Materials and Procedure

All participants completed the symptom and beliefs measures (see below). In addition, the two clinical groups were also administered the Yale–Brown Obsessive–Compulsive Scale [Y-BOCS (15)], which is a clinician-rated 10-item scale. The primary diagnosis was assessed with the Mini international neuropsychiatric interview [MINI PLUS version 5.0 (14)]. The MINI is a structured interview used to diagnose Axis I disorders based on the Diagnostic and Statistical Manual of Mental Disorders [DSM-IV (16)]. Participants reporting relationship-centered and partner-focused symptoms as their primary OCD symptom were diagnosed as ROCD. All other OCD symptom presentations were allocated to the OCD group.

Relationship-centered OC symptoms were assessed with the Relationship Obsessive–Compulsive Inventory [ROCI (5)], a 12-item self-report measure taping into three OC relational dimensions: feelings toward one’s partner (e.g., “I continuously doubt my love for my partner”), partner’s feelings toward oneself (e.g., “I keep asking my partner whether she/he really loves me”), and the rightness of the relationship (e.g., “I check and recheck whether my relationship feels right”). The ROCI scales have been shown to relate to measures of OCD symptoms, anxiety, depression, stress, and relationship quality (5). In the current study, the sum of all ROCI items (Cronbach’s α = 0.93) was used as a measure of relationship-centered symptoms.

Partner-focused OC symptoms were assessed with the Partner-Related Obsessive–Compulsive Symptoms Inventory [PROCSI (8)], a 24-item self-report measure of OC symptoms centered on one’s partner perceived flaws in six domains: appearance, morality, sociability, intelligence, emotional stability, and general competence. The PROCSI has been found to be associated with measures of relationship-centered and general OC symptoms, anxiety, depression, stress, and relationship quality (8). The 24 items were averaged to create a total score of partner-focused symptoms (Cronbach’s α = 0.95).

Severity of OCD symptoms among ROCD and OCD clients was assessed with the clinician administered Yale–Brown Obsessive–Compulsive Scale [Y-BOCS (15)], a 10-item scale that assesses time/frequency, interference in functioning, distress, resistance attempts, and degree of control related to the clients primary obsessions and compulsion. OCD symptom level was assessed through the Obsessive–Compulsive Inventory [OCI-R (17)], an 18-item self-report questionnaire assessing OCD symptoms. In the current study, we used the sum of all OCI-R items (Cronbach’s α = 0.86) as a measure of general OC symptoms.

Depression was assessed through the depression scale of the short version of the Depression Anxiety Stress Scales [DASS (18)], a self-report questionnaire listing negative emotional symptoms. The seven items were averaged to create a depression measure (Cronbach’s α = 0.90).

Obsessive–compulsive disorder-related beliefs were assessed through the short-form of the Obsessive Beliefs Questionnaire (19), a 20-item abbreviated version of the 44-item Obsessive Beliefs Questionnaire-Revised (11). This measure covers four belief domains represented by five items each: (1) Inflated Responsibility, (2) Threat Overestimation, (3) Perfectionism/Intolerance of uncertainty, and (4) Importance/Control of Thoughts. The items of each scale were averaged to create subscale scores (Cronbach’s α ranging from 0.76 to 0.85), and all 20 items were averaged to create a total score of OCD-related beliefs (Cronbach’s α = 0.92).

Finally, maladaptive relational beliefs were assessed through the Relationship Catastrophization Scale (RECATS; see Table A1 in Appendix for the full scale), an 18-item self-report measure designed to tap into three relational belief domains represented by six items each, including: (1) overestimation of the negative consequences of being alone, (2) overestimation of the negative consequences of separating with one’s partner, and (3) overestimation of the negative consequences of being in the wrong relationship.

The RECATS was subjected to confirmatory factor analysis with an independent sample of 218 community participants (50.5% male, 76.6% in a relationship, Mean age = 39.48, Mean relationship length = 12.29 years), which supported the hypothesized three-factor structure (CPI = 0.943, RMSEA = 0.065, SRMR = 0.056; see Table A1 in Appendix for factor loadings). In the current sample, the three subscales demonstrated acceptable internal consistency (Cronbach’s α values ranging from 0.79 to 0.87), as did the scale as a whole (Cronbach’s α = 0.86). Hence, subscale scores and a total score were created by averaging the relevant items. As would be expected, the subscale scores were significantly and positively correlated with each other (*r*s ranging between 0.24 and 0.49).

## Results

### Correlations between Study Variables

Table 1 displays correlations between the four symptom measures (ROCI total, PROCSI total, OCI-R total, and DASS Depression), the total score of the short-form OBQ, and the total score of the RECATS. Not surprisingly, positive correlations were found between all symptom measures in the combined clinical cohort (above the diagonal) and in the overall sample (below the diagonal). As expected, the total OBQ score was positively correlated with all symptom measures, whereas the total RECATS score was positively correlated only with the relationship-related symptom measures (i.e., ROCI and PROCSI).

**Table 1:** **Correlations between relationship-centered (ROCI), partner-focused (PROCSI), and general (OCI-R) obsessive–compulsive symptoms, depression symptoms (DASS D), obsessive beliefs (OBQ T), and relationship catastrophization (RECATS T) in the clinical samples (above the diagonal, *n* = 44) and overall sample (below the diagonal, *n* = 72)**.

	1	2	3	4	5	6
ROCI		0.77***	0.28	0.38*	0.41**	0.60***
PROCSI	0.76***		0.32*	0.41**	0.45**	0.62***
OCI-R	0.40***	0.46***		0.38*	0.60***	−0.01
DASS D	0.42***	0.40***	0.47***		0.49**	0.15
OBQ T	0.45***	0.52***	0.68***	0.68***		0.15
RECATS T	0.47***	0.44***	0.18	0.09	0.22	

*All scales are represented by their total scores*.

*p < 0.05.

**p < 0.01.

***p < 0.001.

### Group Differences in Symptoms

A multivariate analysis of variance (MANOVA) was conducted in order to determine whether ROCD clients differed from OCD clients and the general population in their pattern of OC and affective symptoms. The dependent variables in the analysis were relationship-centered (ROCI total score), partner-focused (PROCSI total score), and general OC symptoms (OCI-R total score), and depression symptoms (DASS Depression subscale).

As expected, the test revealed a significant multivariate effect [*F*(8,130) = 4.59, *p* < 0.001, Λ = 0.61]. Moreover, univariate *post hoc* analyses revealed significant group differences in all symptom measures (see Table 2). Pair-wise comparisons showed that ROCD clients reported more severe relationship-centered and partner-focused symptoms than both OCD clients and community controls, with no differences emerging between the latter two groups. In addition, both ROCD clients and OCD clients reported more severe general OC symptoms than community controls, with no differences emerging between the ROCD and OCD groups. Finally, ROCD clients reported more severe depression symptoms than community controls, with OCD clients scoring higher than the community but not differing significantly from either group. Importantly, the results regarding OC symptoms were not altered when depression was entered as a covariate. Moreover, none of the results were moderated by relationship status, and relationship status was not related to relationship-centered or partner-focused symptom severity in any of the groups. Overall, these findings suggest that ROCD clients experience more severe relationship-related OC symptoms than other OCD clients. ROCD clients, however, also show disabling levels of other OC symptoms and of affective symptoms.

**Table 2:** **Group means, SD, and differences in relationship-centered (ROCI), partner-focused (PROCSI), and general (OCI-R) obsessive–compulsive symptoms, and in depression symptoms (DASS D)**.

	ROCD		OCD		Community		Omnibus	*d*_1vs2_	*d*_1vs3_	*d*_2vs3_
	*M*	SD	*M*	SD	*M*	SD	*F*			
ROCI	2.13_a_	0.80	1.12_b_	0.84	0.89_b_	0.80	14.92***	1.22	1.55	0.28
PROCSI	1.30_a_	0.70	0.81_b_	0.67	0.65_b_	0.65	5.81**	0.73	0.92	0.25
OCI-R	25.62_a_	12.75	21.05_a_	9.50	14.00_b_	8.30	8.17***	0.41	1.08	0.79
DASS D	1.08_a_	0.90	0.90_ab_	0.73	0.52_b_	0.54	3.99*	0.23	0.76	0.59

*Within each row, means that do not share subscripts differ significantly (*p* < 0.05; Tukey HSD)*.

*p < 0.05.

**p < 0.01.

***p < 0.001.

A separate MANOVA was conducted in order to compare the ROCD and OCD groups on severity of obsession and compulsion symptoms as assessed through the Y-BOCS. As expected, there were no significant differences at the multivariate level or the univariate level (all *F*s < 1). Symptom severity was remarkably similar in the ROCD group (*M*s = 11.33, 11.13, and 22.47 for obsessions, compulsions, and total score, respectively) and the OCD group (*M*s = 11.57, 11.52, and 23.10 for obsessions, compulsions, and total score, respectively). These findings suggest ROCD and OCD clients show comparable interference in functioning, distress, resistance attempts, and degree of control related to their primary obsessions and compulsion.

### Group Differences in OCD-Related Beliefs

A third MANOVA was conducted in order to determine whether ROCD clients differ from OCD clients and the general population in their pattern of OCD-related beliefs. The dependent variables in the analysis were the four subscales of the short-form OBQ. As expected, the test revealed a significant multivariate effect [*F*(8,130) = 4.52, *p* < 0.001, Λ = 0.62]. Univariate *post hoc* analyses revealed significant group differences in all belief domains (see Table 3). Pair-wise comparisons showed that ROCD clients were more prone to attribute importance to thoughts and have an inflated sense of responsibility than both OCD clients and community controls. In addition, ROCD clients were more prone to threat overestimation and perfectionism than community controls, but were not significantly more prone to these beliefs than OCD clients. OCD clients were higher than community controls in their tendency to attribute importance to thoughts. Overall, these findings suggest that the belief system found to be related to OCD characterizes ROCD as well, and in some instances such beliefs may be even more pronounced among ROCD clients.

**Table 3:** **Group means, SD, and differences in OBQ over estimation of threat (OBQ OT), perfectionism/intolerance of uncertainty (OBQ PU), importance of thoughts (OBQ IT), and inflated responsibility (OBQ IR) subscales**.

	ROCD		OCD		Community		Omnibus	*d*_1vs2_	*d*_1vs3_	*d*_2vs3_
	*M*	SD	*M*	SD	*M*	SD	*F*
OBQ OT	3.80_a_	1.65	3.29_ab_	1.57	2.67_b_	1.25	3.64*	0.32	0.77	0.44
OBQ PU	4.22_a_	1.48	3.45_ab_	1.56	3.08_b_	1.14	4.24*	0.50	0.86	0.28
OBQ IT	4.19_a_	1.46	2.94_b_	1.20	2.11_c_	0.98	18.30***	0.94	1.68	0.76
OBQ IR	4.26_a_	1.71	3.27_b_	1.27	3.01_b_	1.15	5.45**	0.66	0.86	0.22

*Within each row, means that do not share subscripts differ significantly (*p* < 0.05; Tukey HSD)*.

*p < 0.05.

**p < 0.01.

***p < 0.001.

### Group Differences in Maladaptive Beliefs about Relationships

The final MANOVA was conducted in order to determine whether ROCD clients differ from OCD clients and the general population in their pattern of maladaptive beliefs about relationships. The dependent variables in the analysis were the three subscales of the RECATS. Once again, the test revealed a significant multivariate effect [*F*(6,122) = 2.20, *p* < 0.05, Λ = 0.81]. Univariate *post hoc* analyses revealed significant group differences in two of the three relational belief domains (see Table 4). Pair-wise comparisons showed that ROCD clients were more prone to overestimate the negative consequences of being in the wrong relationship than both OCD clients and community controls. ROCD clients were also more prone to overestimate the negative consequences of being alone compared with community controls, but not compared with OCD clients. Although ROCD clients in the sample were more prone to overestimate the negative consequences of separating with one’s partner than OCD clients and community controls, these differences failed to reach significance. Importantly, OCD clients did not differ than community controls in any of the relational belief domains. Overall, these findings suggest that in addition to endorsing obsessive beliefs related to OCD in general, ROCD clients endorse an additional and unique set of maladaptive beliefs about relationships.

**Table 4:** **Group means, SD, and differences in RECATS consequences of being alone (REC A), separating from partner (REC S), and being in the wrong relationship (REC R) subscales**.

	ROCD		OCD		Community		Omnibus	*d*_1vs2_	*d*_1vs3_	*d*_2vs3_
	*M*	SD	*M*	SD	*M*	SD	*F*
REC A	4.23_a_	1.55	3.44_ab_	1.34	3.31_b_	1.05	3.10*	0.55	0.69	0.11
REC S	3.49_a_	1.36	3.04_a_	1.25	2.82_a_	1.29	1.54	0.34	0.51	0.18
REC R	4.88_a_	1.17	3.64_b_	1.23	3.61_b_	1.46	6.13**	1.03	0.96	0.02

*Within each row, means that do not share subscripts differ significantly (*p* < 0.05; Tukey HSD)*.

*p < 0.05.

**p < 0.01.

## Discussion

Relationship obsessive–compulsive disorder is an understudied presentation of OCD that poses specific challenges for CBT-based psychological interventions for OCD. Previous research with non-clinical cohorts has shown that ROCD symptoms are associated with a variety of negative personal and dyadic consequences. The present study is the first systematic comparison of clients diagnosed with ROCD with OCD clients and community controls on functioning level and various symptoms and maladaptive beliefs.

Supporting our interview-based group assignation, our results indicate ROCD clients showed higher levels of relationship-centered and partner-focused OC symptoms than did both the OCD clients and community controls. ROCD clients also reported higher levels of depression than community control, but not more than their OCD counterparts. Finally, ROCD and OCD clients showed similar levels of interference in functioning, distress, resistance attempts, and degree of control relating to their primary obsessional concerns as measured by the Y-BOCS. These findings suggest that ROCD clients show similar levels of disability as other OCD clients.

Supporting the conceptualization of ROCD as an additional dimension of OCD (2), ROCD symptoms showed moderate-size correlations with other OCD symptoms and both clinical groups showed higher scores on OCD symptoms than community controls. Like other OCD symptom dimensions [e.g., scrupulosity (20)], individuals with ROCD may show elevations in a variety of other OCD symptom dimensions. Finally, ROCD and OCD clients showed remarkably similar level of distress, resistance attempts, and degree of control relating to their primary obsessional concerns. Although, OCD symptom presentations may vary markedly in their theme of preoccupation, all maintain the hallmark of recurring pattern of distressing obsessions that beget compulsions, which in turn beget further obsessions, and so forth.

Recent cognitive models highlight both common and specific maintaining factors of particular OCD presentations (21). For instance, cognitive models of OCD propose that inflated responsibility beliefs and beliefs that thoughts can and should be controlled are important and specific to OCD [e.g., Ref. (22)]. Other beliefs such as perfectionism and overestimation of threat are important in the dynamics of OCD, but do not seem to be specific to this disorder (10, 11, 23). Consistent with this, both the ROCD and OCD patient groups in our sample displayed small-to-large effect size differences on OCD-related beliefs compared to the non-clinical control group with *post hoc* tests revealing the importance/control of thoughts subscale showing the greatest effect size in differentiating between groups.

Our results also suggest that ROCD clients may be more prone than their OCD counterparts to endorse inflated responsibility beliefs and to attribute increased importance to thoughts and to their control. ROCD symptoms inherently involve a decision pertaining to a significant other (i.e., the partner). As such, inflated responsibility beliefs may intensify negative emotional responses (e.g., guilt and self-blame) following relationship-related doubts, increasing distress and dysfunctional responses. Similarly, attributing importance to thoughts and their control may increase vigilance to negative thoughts about one’s partner or the relationship increasing relationship doubts and maladaptive compulsive behaviors (e.g., checking and comparing). Greater endorsement of the importance of thoughts also may reflect that relationship OCD leads to high levels of monitoring of internal states.

Overall, ROCD clients in our sample showed higher levels of relationship maladaptive beliefs than both control groups. Specifically, ROCD clients perceived the ramifications of being in the wrong relationship more negatively than OCD clients and controls. ROCD clients also estimated the negative consequences of being alone significantly higher than community controls. Indeed, ROCD clients often describe catastrophic scenarios of being forever trapped in unsatisfying, distressing relationships, and at the same time, fearing being alone. Such beliefs may maintain ROCD symptoms by increasing the likelihood of appraising common relationship experiences (e.g., feeling bored or stressed in the company of the partner) as suggesting relational incompatibility. Moreover, the elevated endorsement beliefs about the consequences of being alone would work in opposition to fears of being in the wrong relationship, as such, the two beliefs would catch the individual in a “double bind,” simultaneously doubting their relationship but also fearing being alone. Finally, the lack of differences on the scale assessing negative consequences of separation suggests that it is not the immediate cost of the break up that is important, but rather the perceived negative long-term consequences of being alone vs. being unhappy together with a partner.

Our results point to the involvement of several factors, some specific to ROCD (i.e., relationship maladaptive beliefs) and some common with other OC dimensions (i.e., OCD-related beliefs) in the maintenance of this particular theme of OC symptoms. Additional relationship-related factors such as fear of abandonment and over-reliance of self-worth on the relationship or relationship partner (4, 6) may play an important role in development and maintenance of ROCD symptoms. Moreover, ROCD symptoms occur in additional relational contexts such as ­parent–child and God–individual (2, 3). Future investigations may assess whether ROCD symptoms constituted a subcategory of OCD symptoms with specific vulnerability factors and treatment targets. Such studies may include provoking ROCD symptoms in one context [e.g., romantic; see Ref. (6) for such a methodology] and assessing the relative increase of OCD symptoms (e.g., contamination) and ROCD symptom in different relational contexts (e.g., God–Individual).

Future investigations of ROCD may also consider a number of potential limitations of the current study. First, the cross-sectional nature of the study precludes any causal conclusions. Future studies with ROCD populations may wish to track these beliefs through treatment, to see whether there is belief change preceding clinical change. Second, the link between relationship-related beliefs and other aspects noted in studies on ROCD [e.g., attachment insecurities and self-beliefs and sensitivities, see Ref. (2) for a review] should be examined, as well as the way in which OCD-beliefs and relationship beliefs interact. In this context, larger ROCD and OCD groups would also allow examination of differences between particular symptom dimensions (e.g., relationship vs. scrupulosity). Thus, a replication of the findings using larger groups is important.

In ROCD treatment, our findings highlight the importance of challenging catastrophic beliefs regarding relationships including overestimation of the negative consequences of staying in relationships and the negative consequences of being alone in addition to OCD-related beliefs. Other extensions to CBT may also be useful, such as exploring contingencies of self-worth on relational aspects, and attachment worries and anxieties, particularly fear of abandonment [see Ref. (24) and (25) for a description]. Finally, the client may need to be taught social skills for relationships such as communication and conflict resolution.

In conclusion, this study marks the first systematic clinical study of individuals with relationship-centered OCD symptoms, in comparison to both clinical (OCD), and non-clinical controls. Results lend credence to the view of ROCD as a distressing dimension of OCD – with levels of symptomatology equal to or higher to other OCD themes. The study also highlights the importance of negative beliefs within the disorder. Hopefully, these findings will help inform treatments and general awareness of this debilitating condition.

## Ethics Statement

This study was approved by The Interdisciplinary Center (IDC) Herzliya Research Ethics Commitee. All participants were informed of their rights and completed an informed consent form in accordance with university IRB standards.

## Author Contributions

GD and DD developed the study concept and design. Data collection was performed by EN and DD, while data analysis and interpretation were performed by GD, OS, and RM. The paper was drafted by all authors. All authors approved the final version of the paper for submission.

## Conflict of Interest Statement

The authors declare that the research was ­conducted in the absence of any commercial or financial relationships that could be construed as a potential conflict of interest.

## Appendix

**Table A1:** **Relationship Catastrophization Scale (RECATS): items and factor loadings**.

**Overestimation of the consequences of being alone**		
1.	Being without a partner would cause great pain to me and everyone around me	0.79
2.	The thought of going through life without a partner scares me to death	0.76
3.	“It is not good that the man should be alone” is a verse I live my life by	0.77
4.	If there is something I do not wish anyone, it is being alone in the world, without a relationship	0.81
5.	I believe there is nothing more important than romantic relationships	0.79
6.	For me, living without a romantic relationship is not living at all	0.68
**Overestimation of the consequences of separation**		
7.	I would prefer almost anything over having to deal with the consequences of breaking up with my partner	0.74
8.	I think breaking up with a partner is one of the worst things that can happen to anyone	0.74
9.	I am convinced that breaking up with my partner might cause both of us irreparable damage	0.75
10.	As far as I am concerned, there is nothing harder than dealing with a break-up	0.75
11.	I feel I can not take back my romantic decisions	0.65
12.	It is almost impossible for me to leave a romantic relationship	0.75
**Overestimation of the consequences of being in the wrong relationship**		
13.	For me, being in an imperfect relationship is like betraying myself	0.56
14.	I think bad romantic decisions almost always cost dearly	0.75
15.	In my opinion, a romantic relationship that does not always feel right is probably a destructive relationship	0.60
16.	I believe that making the wrong romantic choice is often a terrible thing	0.70
17.	I believe that being in the wrong relationship almost always leads to a wasted life	0.64
18.	I believe that making the wrong romantic decision would put me on a path of great misery	0.75
